# Tunable third harmonic generation based on high-Q polarization-controlled hybrid phase-change metasurface

**DOI:** 10.1515/nanoph-2024-0113

**Published:** 2024-06-03

**Authors:** Yi Tao, Dong-Qin Zhang, Zhong-Wei Jin, Gui-Ming Pan, Jian-Yuan Qin, Zhi Hong, Bin Fang, Fang-Zhou Shu

**Affiliations:** Department of Physics, 92270 China Jiliang University, Hangzhou, 310018, China; National Laboratory of Solid State Microstructures, and School of Physics, and Collaborative Innovation Center of Advanced Microstructures, Nanjing University, Nanjing, 210093, China; Centre for Terahertz Research, 92270 China Jiliang University, Hangzhou, 310018, China

**Keywords:** metasurface, phase-change material, third harmonic generation, bound states in the continuum

## Abstract

Dielectric metasurfaces have made significant advancements in the past decade for enhancing light–matter interaction at the nanoscale. Particularly, bound states in the continuum (BICs) based on dielectric metasurfaces have been employed to enhance nonlinear harmonic generation. However, conventional nonlinear metasurfaces are typically fixed in their operating wavelength after fabrication. In this work, we numerically demonstrate tunable third harmonic generation (THG) by integrating a dielectric metasurface with the phase-change material Ge_2_Sb_2_Te_5_ (GST). The hybrid phase-change metasurface can support two BICs with different electromagnetic origins, which are transformed into two high-Q quasi-BICs through the introduction of structural asymmetry. The two quasi-BICs are selectively excited by controlling the polarization of incident light, and their wavelengths are tunable due to the phase transition of GST. Notably, the efficiency of THG is significantly enhanced at the fundamental wavelengths corresponding to the two quasi-BICs, and the operating wavelength for THG enhancement can be dynamically tuned through the GST phase transition. Furthermore, the wavelength of THG enhancement can be further tuned by manipulating the polarization of pump light. Additionally, a high-Q analog of electromagnetically induced transparency is numerically achieved through the interaction between a low-Q Mie resonance and a quasi-BIC mode, which also improves the THG efficiency. The high-Q polarization-controlled hybrid phase-change metasurface holds promise for applications in dynamically tunable nonlinear optical devices.

## 1 Introduction

Metasurfaces have garnered considerable interest in the last decade for their versatile and efficient manipulation of electromagnetic wave [1], [2]. They employ the resonant modes in subwavelength metallic or dielectric nanostructures to control the amplitude, phase, and polarization of far-field. Additionally, these resonant modes possess the capability of enhancing electromagnetic near-field at the nanoscale, which is advantageous for nonlinear optics applications [3], [4], [5], [6]. Although early researches focused on surface plasmon resonances in metallic nanostructures for nonlinear harmonic enhancement, their performances were limited by the inherent ohmic losses of metals [3], [4]. In recent years, dielectric metasurfaces have emerged as a highly promising platform for nonlinear optics [5], [6]. Dielectric nanostructures support a variety of Mie resonant modes that can confine electromagnetic fields within the structures [7], [8]. Enhanced nonlinear harmonic generation has been achieved in dielectric metasurfaces by utilizing magnetic dipole mode [9], anapole state [10], electromagnetically induced transparency (EIT) [11], [12], and bound states in the continuum (BICs) [13], [14], [15], [16], [17], [18]. Particularly, BICs have garnered significant attention due to their strong light–matter interaction capabilities [19], [20], [21]. BICs are localized states without any radiation but exist within the radiation continuum. Dielectric metasurfaces offer an effective platform for the creation of BICs [19], [20], [21]. Ideal BICs possess infinite quality (Q) factors and are unavailable by incident light. However, by introducing small structural perturbations, BICs are transformed into high-Q quasi-BICs that can be directly coupled with incident light. Quasi-BICs can provide extreme electromagnetic field confinement, which has been harnessed to enhance nonlinear conversion efficiency [13], [14], [15], [16], [17], [18].

While there has been extensive research on nonlinear metasurfaces, the operating wavelengths for efficient nonlinear harmonic generation are typically fixed once the samples are fabricated. In order to enable efficient nonlinear harmonic generation across different wavelengths, it is often necessary to utilize multiple metasurfaces with diverse structural parameters, leading to elevated fabrication costs. Hence, developing wavelength-tunable nonlinear metasurface is desired in realistic applications. In recent years, active metasurfaces have attracted widespread attention to realize tunable functions [22], [23]. The primary approach for achieving tunable metasurfaces involves the integration of metasurfaces with active materials such as liquid crystals [24], graphene [25], polymers [26], and phase-change materials [27], [28], [29]. Among these active materials, Ge_2_Sb_2_Te_5_ (GST), as a phase-change material, is particularly favored by researchers due to its ultrafast switching speed, thermal stability, and switching robustness characteristics [28], [29]. The significant refractive index variation of GST between amorphous and crystalline phases has been harnessed for the design of various tunable linear optical devices through integration with metasurfaces [30], [31], [32], [33], [34], [35], [36], perovskite [37], and waveguide [38]. Recent studies have explored the nonlinear susceptibilities of GST in both amorphous and crystalline states, indicating its potential for dynamic nonlinear optical devices [39], [40], [41], [42], [43], [44], [45]. Tunable third harmonic generation (THG) has been achieved by combining GST with a Fabry–Perot cavity [39], [40] or metasurfaces [41], [42], [43]. Particularly, multilevel THG intensity modulation has been proposed in all-dielectric phase-change metasurfaces [43]. However, previous works have shown low THG efficiencies, and BICs have not been applied to GST to enhance the nonlinear conversion efficiency. Furthermore, prior demonstrations of tunable THG have been either polarization-insensitive [39], [40], [41] or restricted to a single polarization [42], [43]. Notably, the polarization can serve as a degree of freedom for realizing multifunctional optical devices [46]. Nevertheless, the simultaneous utilization of polarization control and phase transition of GST to achieve multiple THG enhancement has not yet been discussed.

In this work, we propose a high-Q polarization-controlled hybrid phase-change metasurface to realize tunable THG enhancement. The metasurface supports two BICs with magnetic dipole (MD) and electric dipole (ED) origins. By introducing a defect to break structural symmetry, the MD BIC is transformed into the MD quasi-BIC under *x*-polarized incidence, while the ED BIC is converted into the ED quasi-BIC under *y*-polarized incidence. The wavelengths associated with these two quasi-BICs can be dynamically tuned through the phase transition of GST. Moreover, efficient THG signals can be produced at the fundamental wavelengths corresponding to both MD and ED quasi-BICs, and the operating wavelength is tunable based on the GST phase transition. Since the wavelength of MD quasi-BIC is different from that of the ED quasi-BIC, multiple enhanced THG signal can be realized in a single metasurface by manipulating the polarization state of pump light and the phase of GST. Additionally, by adjusting the geometric parameters of the hybrid phase-change metasurface, a high-Q EIT can be achieved through the interaction between the low-Q MD mode and MD quasi-BIC or between the low-Q ED mode and ED quasi-BIC. Furthermore, THG signal is also enhanced at the EIT wavelength and can be dynamically controlled through the GST phase transition. The high-Q polarization-controlled hybrid phase-change metasurface offers a promising platform for developing versatile nonlinear optical devices.

## 2 Structure design and method

**Figure 1:**
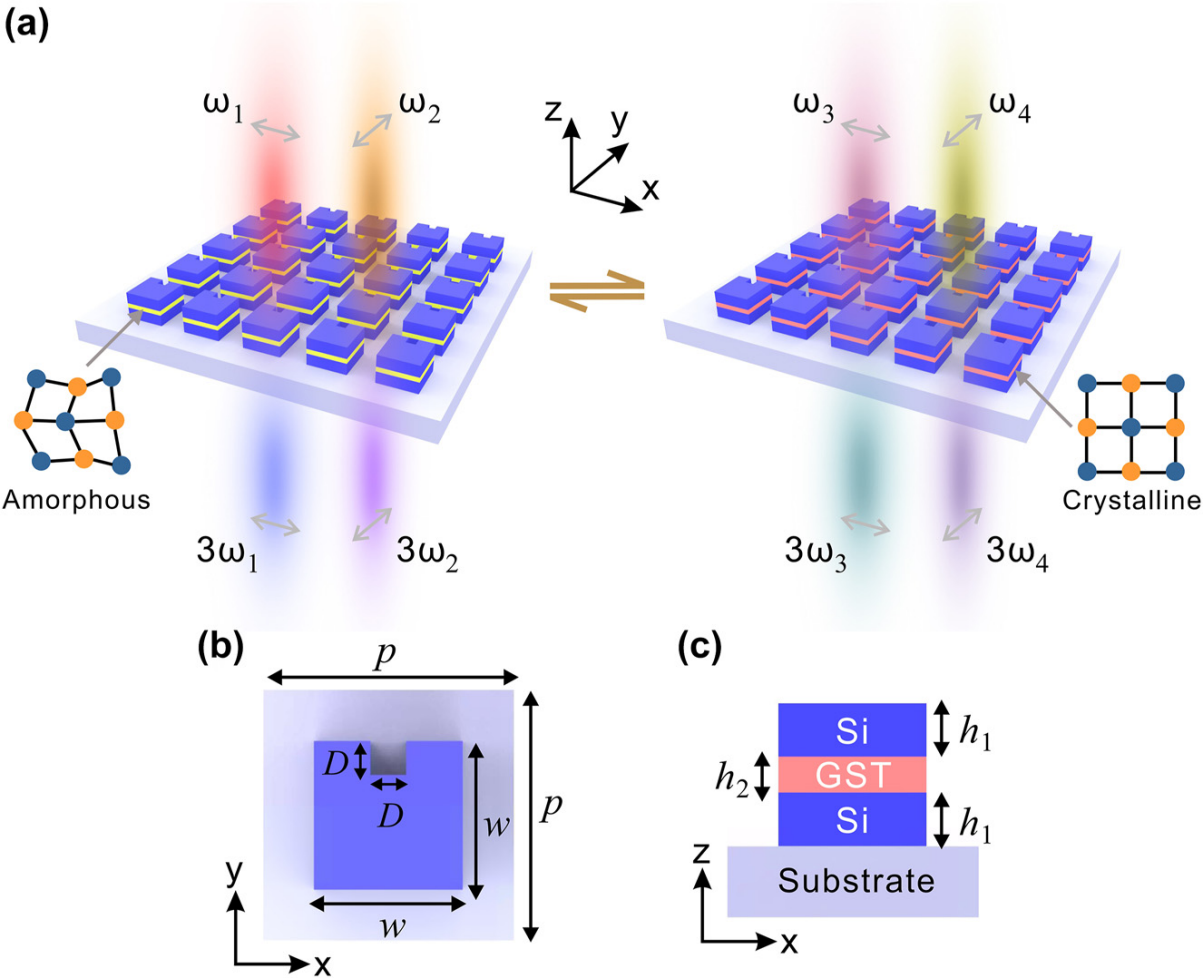
Schematic of the hybrid phase-change metasurface. (a) Schematic of the tunable THG based on the high-Q polarization-controlled hybrid phase-change metasurface. (b) Top and (c) cross-sectional views of a single unit cell.

Dynamically tunable THG based on the high-Q polarization-controlled hybrid phase-change metasurface is schematically illustrated in Figure 1(a). The metasurface is designed using periodic composite nanostructures composed of silicon (Si) and GST. As shown in Figure 1(b) and (c), the GST nanostructure is sandwiched between two Si nanostructures in each unit cell, and the Si-GST-Si nanostructure is formed by removing a small square block in the Si-GST-Si square block. The hybrid Si-GST-Si metasurface has been previously proposed, and tunable ED and MD modes are demonstrated [32]. Compared with previous study with symmetric structures [32], the introduction of defect in this work breaks the in-plane inversion symmetry, resulting in the excitation of two high-Q quasi-BICs with distinct electromagnetic origins and frequencies. One quasi-BIC with frequency of *ω*_1_ is stimulated by *x*-polarized incident light, while another quasi-BIC with frequency of *ω*_2_ is triggered by *y*-polarized incident light. Both Si and GST have large third-order nonlinear susceptibilities [11], [39]. The third-order nonlinear polarization $P\left(3\omega \right)=\varepsilon_{0}\chi^{\left(3\right)}E\left(\omega \right)\cdot E\left(\omega \right)\cdot E\left(\omega \right)$ in Si-GST-Si nanostructures serves as a source to generate the THG, where *ε*_0_ is the vacuum permittivity, *χ* ^(3)^ is the third-order nonlinear susceptibility, and *E*(*ω*) is the electric field. As the electric field within Si-GST-Si nanostructures can be significantly enhanced by the excitation of quasi-BICs, an efficient THG can be achieved based on the hybrid phase-change metasurface. When the pump light is polarized along the *x*-direction at the fundamental frequency of *ω*_1_, an enhanced THG signal is produced at the frequency of 3*ω*_1_. However, changing the pump light polarization to the *y*-direction at the fundamental frequency of *ω*_2_ results in an enhanced THG at the frequency of 3*ω*_2_. Consequently, by manipulating the polarization state of pump light, frequency-controlled THG enhancement can be achieved. Since the refractive index of GST can be tuned through its phase transition, these quasi-BICs shift to other frequencies (*ω*_3_ and *ω*_4_) when GST transitions from the amorphous phase to the crystalline phase. Correspondingly, adjusting the fundamental frequency of the pump light to *ω*_3_ or *ω*_4_ under *x*- or *y*-polarized incidence leads to enhanced THG signals at the frequency of 3*ω*_3_ or 3*ω*_4_, respectively. This approach enables the realization of multiple enhanced THG in a single metasurface by controlling the phase of GST and the polarization state of pump light, as depicted in Figure 1(a).

In order to validate the above analysis, numerical simulations were performed utilizing the commercial software COMSOL Multiphysics. In the simulations, we set periodic boundary conditions in the *x*- and *y*-direction while perfect matching layers in the *z*-direction. A plane wave with *x*- or *y*-polarization was incident along the *z*-direction. The permittivities of silicon and silica were obtained from the literature [47], whereas the permittivities of GST in amorphous and crystalline phases were taken from the literature [48]. Additionally, GST has multiple intermediate phases during the phase transition, and the permittivities of these intermediate phases can be computed based on the Lorentz–Lorentz relation [31]:

$$\frac{\varepsilon_{i}-1}{\varepsilon_{i}+2}=\text{s}\frac{\varepsilon_{c}-1}{\varepsilon_{c}+2}+\left(1-s\right)\frac{\varepsilon_{a}-1}{\varepsilon_{a}+2}$$

where *ε*_*a*_, *ε*_*i*_, and *ε*_*c*_ denote the permittivities associated with the amorphous, intermediate, and crystalline phases of GST, respectively, with *s* representing the crystallinity of GST. The third-order nonlinear susceptibility of Si ( $\chi_{Si}^{\left(3\right)})$ was chosen as 2.45 × 10^−19^ m^2^/V^2^ [17], while the third-order nonlinear susceptibility of silica was negligible. The third-order nonlinear susceptibility of GST ( $\chi_{\mathrm{GST}}^{\left(3\right)})$ in amorphous and crystalline phases were set to 8.25 × 10^−19^ and 9.9 × 10^−18^ m^2^/V^2^, respectively, as done in the reference [41]. The third-order nonlinear susceptibility of GST in the semicrystalline phase (crystallinity of 50 %) was selected to be 3.5 times greater than that in the amorphous phase, as indicated in the reference [40]. In nonlinear simulations, the linear electric fields inside Si structure (*E*_*Si*_(*ω*)) and GST structure (*E*_*GST*_(*ω*)) were firstly calculated at the fundamental wavelength. Then, the third-order nonlinear polarization was computed according to $P\left(3\omega \right)=\varepsilon_{0}\chi_{Si}^{\left(3\right)}E_{Si}\left(\omega \right)\cdot E_{Si}\left(\omega \right)\cdot E_{Si}\left(\omega \right)+\varepsilon_{0}\chi_{\mathrm{GST}}^{\left(3\right)}E_{\mathrm{GST}}\left(\omega \right)\cdot E_{\mathrm{GST}}\left(\omega \right)\cdot E_{\mathrm{GST}}\left(\omega \right)$ , which was employed as a source to obtain the THG. The conversion efficiency of THG can be calculated through *η*_*THG*_ = *P*_*THG*_/*P*_Pump_ where *P*_*THG*_ denotes the THG power and *P*_Pump_ represents the pump power. The *P*_*THG*_ was calculated by integrating the Poynting vector of the THG in the transmitted plane, while the *P*_Pump_ was calculated by *P*_Pump_ = *I*_0_⋅*A*, where *I*_0_ was the pump intensity and *A* was the area of unit cell. The pump laser intensity of the incident continuous plane wave was chosen as 1 MW/cm^2^. The geometric parameters including the period (*P*), side length (*W*), Si thickness (*h*_1_), and GST thickness (*h*_2_) were held constant at 1,400, 830, 150, and 50 nm, respectively, while the side length of the defect (*D*) was varied in the simulations. These geometric parameters were chosen in order to generate BICs in the wavelength range from 2.4 to 3.6 μm, where the absorption loss of GST was low for both amorphous and crystalline phases [48].

## 3 Results and discussion

**Figure 2:**
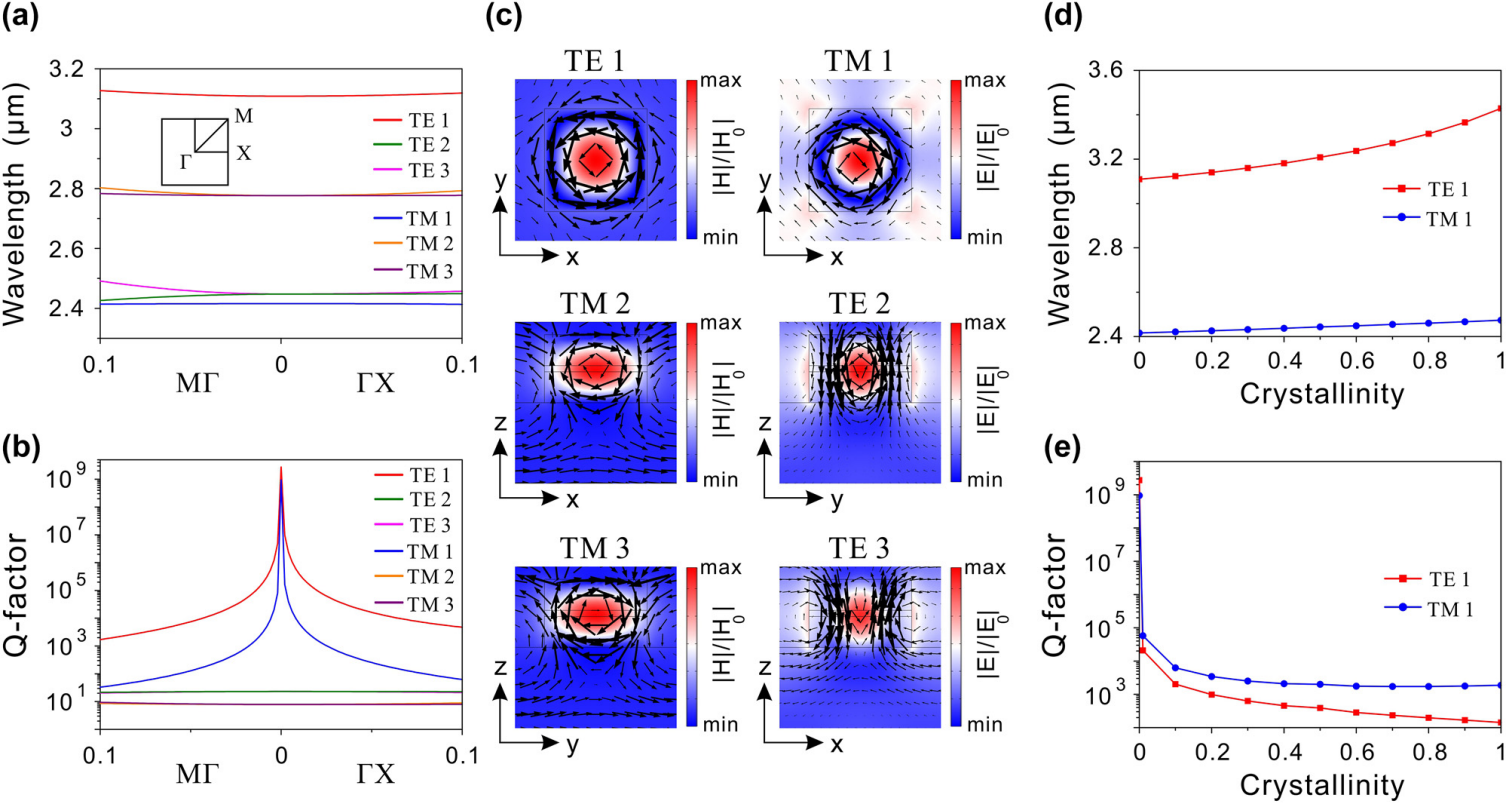
Modal properties of the hybrid phase-change metasurface without defect. (a) Band diagrams of the phase-change metasurface without defect for GST in the amorphous phase. (b) Q-factors of three TE modes and three TM modes in both MΓ and ΓX directions. (c) Electromagnetic field distributions of these TE and TM modes at the Γ point. In the left three images, the colors indicate the amplitude of the magnetic field, and the arrows represent the electric field vectors. In the right three images, the colors represent the amplitude of electric field, and the arrows indicate the magnetic field vectors. (d) Wavelengths and (e) Q-factors of the TE 1 and TM 1 modes at the Γ point as a function of the crystallinity of GST.

Firstly, we investigate the modal properties of the hybrid phase-change metasurface in the absence of defect through the eigenmode analysis. The band diagram in Figure 2(a) illustrates all transverse electric (TE)-like and transverse magnetic (TM)-like modes in the considered wavelength range for GST in its amorphous phase. Figure 2(b) and (c) depict the corresponding Q-factor and electromagnetic field distribution for each mode, respectively. It can be found that the TM 2 and TM 3 modes are degenerate at the Γ point with low Q-factors. The electromagnetic field distribution of TM 2 and TM 3 reveals the alignment of the magnetic field along the *y*- or *x*-direction, while a circular electric field is formed in the *XZ* or *YZ* plane, indicating that these two modes are *y*- and *x*-direction MD modes, respectively. Similarly, the TE 2 and TE 3 modes also display degeneracy at the Γ point, characterized by low-Q *x*- and *y*-direction ED modes, respectively. However, the Q-factors of the TE 1 and TM 1 modes exhibit a rapid increase near the Γ point, approaching infinity at the Γ point, which are clear features of BICs [19], [20], [21]. The electromagnetic field distribution shows that the TE 1 mode features a circular electric field in the *XY* plane and a magnetic field in the *z*-direction, indicating that this mode is mainly dominated by the *z*-direction MD mode. In contrast, the TM 1 mode presents a circular magnetic field in the *XY* plane and an electric field in the *z*-direction, suggesting that it is predominated by the *z*-direction ED mode. Consequently, the hybrid phase-change metasurface can support two BIC modes with *z*-direction ED and MD origins. The wavelengths and Q-factors of the TE 1 and TM 1 modes during the phase transition of GST were also calculated and presented in Figure 2(d) and (e), respectively. As the crystallinity increases, both TE 1 and TM 1 modes undergo a redshift, due to the increase in the refractive index of GST. Given the sensitivity of BICs to absorption loss in composite materials, the Q-factors of both modes decrease as GST is gradually transmitted into the crystalline phase, owing to the increased absorption loss. As a result, tunable BICs can be achieved utilizing the hybrid phase-change metasurface.

**Figure 3:**
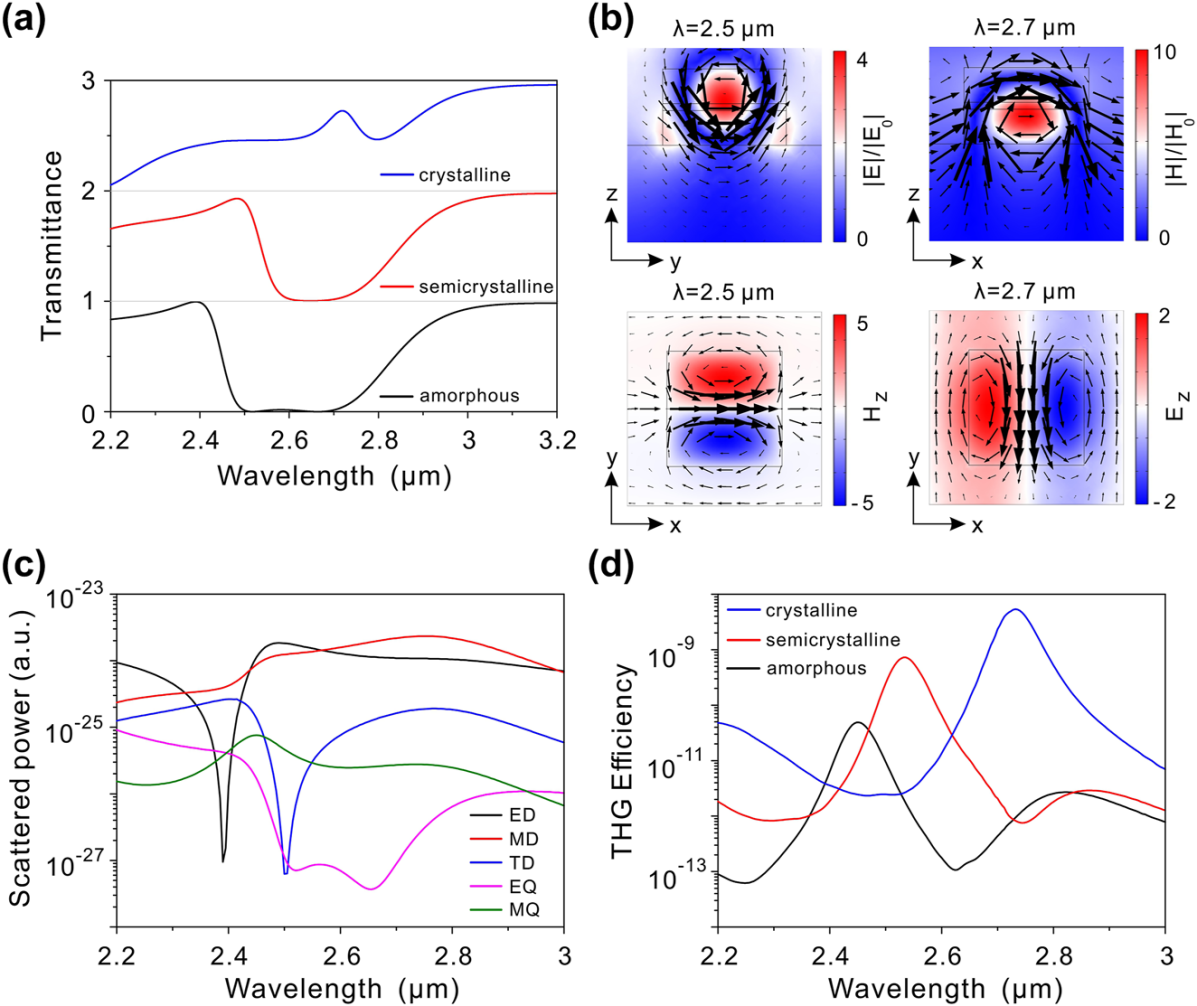
Optical properties of the phase-change metasurface without defect. (a) Transmission spectra of the hybrid phase-change metasurface without defect for GST in amorphous, semicrystalline, and crystalline phases. (b) Electromagnetic field distributions at 2.5 and 2.7 μm for GST in the amorphous phase. In the upper left panel, the color presents the amplitude of the electric field, and the arrows represent the magnetic field vectors. In the upper right panel, the color represents the amplitude of magnetic field, and the arrows indicate the electric field vectors. In the lower left panel, the color presents the *z*-component magnetic field (*Hz*), and the arrows represent the electric field vectors. In the lower right panel, the color represents the *z*-component electric field (*Ez*), and the arrows indicate the magnetic field vectors. (c) Cartesian scattered powers of multipole moments for GST in the amorphous phase. (d) THG efficiency as a function of the pump wavelength at different phases of GST.

The transmission spectra of the hybrid phase-change metasurface without defect at various crystallinities of GST under *x*-polarized incidence are depicted in Figure 3(a). In the amorphous phase, two dips are observed around 2.5 and 2.7 μm, closely positioned, resulting in low transmittance in the wavelength range of 2.5–2.7 μm. To elucidate the origins of these dips, the electric and magnetic field distributions at the two resonant wavelengths were calculated, as plotted in Figure 3(b). At the wavelength of 2.5 μm, the electric field is oriented along the *x*-direction and localized within the structure, while the magnetic field forms a loop in the *YZ* plane, indicating excitation of the ED mode at this wavelength. Conversely, at 2.7 μm, the electric field forms a loop in the *XZ* plane and the magnetic field is distributed along the *y*-direction within the structure, indicating excitation of the MD mode at this wavelength. This modal analysis was further supported by calculating the Cartesian multipolar scattering powers, considering five main contributions from ED, MD, toroidal dipole (TD), electric quadrupole (EQ), and magnetic quadrupole (MQ) moments. As observed in Figure 3(c), the scattered power of the ED moment is higher than that of other multipole moments at the wavelength of 2.5 μm, corroborating the earlier electromagnetic field analysis of this dip. Additionally, the scattered power of the MD moment predominates around 2.7 μm, supporting the inference that the second dip is attributed the MD mode. Upon the phase transition of GST from the amorphous phase to the crystalline phase, both dips undergo a redshift due to the increased refractive index of GST, as shown in Figure 3(a). The wavelength shift of the ED resonance is much larger than that of the MD resonance, and the two dips gradually merge into a single dip as the crystallinity increases. Consequently, tunable ED and MD modes can be realized based on the Si-GST-Si nanostructures.

As discussed above, the electromagnetic fields are enhanced within the Si-GST-Si nanostructures through the excitation of ED and MD modes, which can be employed to enhance the THG conversion efficiency. Figure 3(d) shows the calculated THG efficiencies of the hybrid phase-change metasurface with different phases of GST. In the amorphous phase of GST, two distinct peaks are observed at 2.45 and 2.82 μm, close to the ED and MD wavelengths previously discussed. Therefore, enhanced THG is attributed to the excitation of the ED and MD modes. Since the electric field enhancement of the ED mode is stronger than that of the MD mode, the hybrid phase-change metasurface exhibits a higher THG efficiency at fundamental wavelength of 2.45 μm. For comparison, the THG efficiency for an unpatterned Si-GST-Si film is around 10^−14^, when the fundamental wavelength is 2.45 μm and GST is in the amorphous phase. Consequently, the THG efficiency is enhanced by a factor of 10^3^ relative to the Si-GST-Si thin film, based on the excitation of the ED resonance. Furthermore, with the amorphous to crystalline transition, the peak wavelength of THG efficiency shifts from 2.45 to 2.73 μm, due to the redshift of the ED mode. Meanwhile, the THG peak efficiency rises owing to the increase in the third-order nonlinear susceptibility of GST [40]. As a result, wavelength-tunable THG enhancement can be achieved through the phase transition of GST.

**Figure 4:**
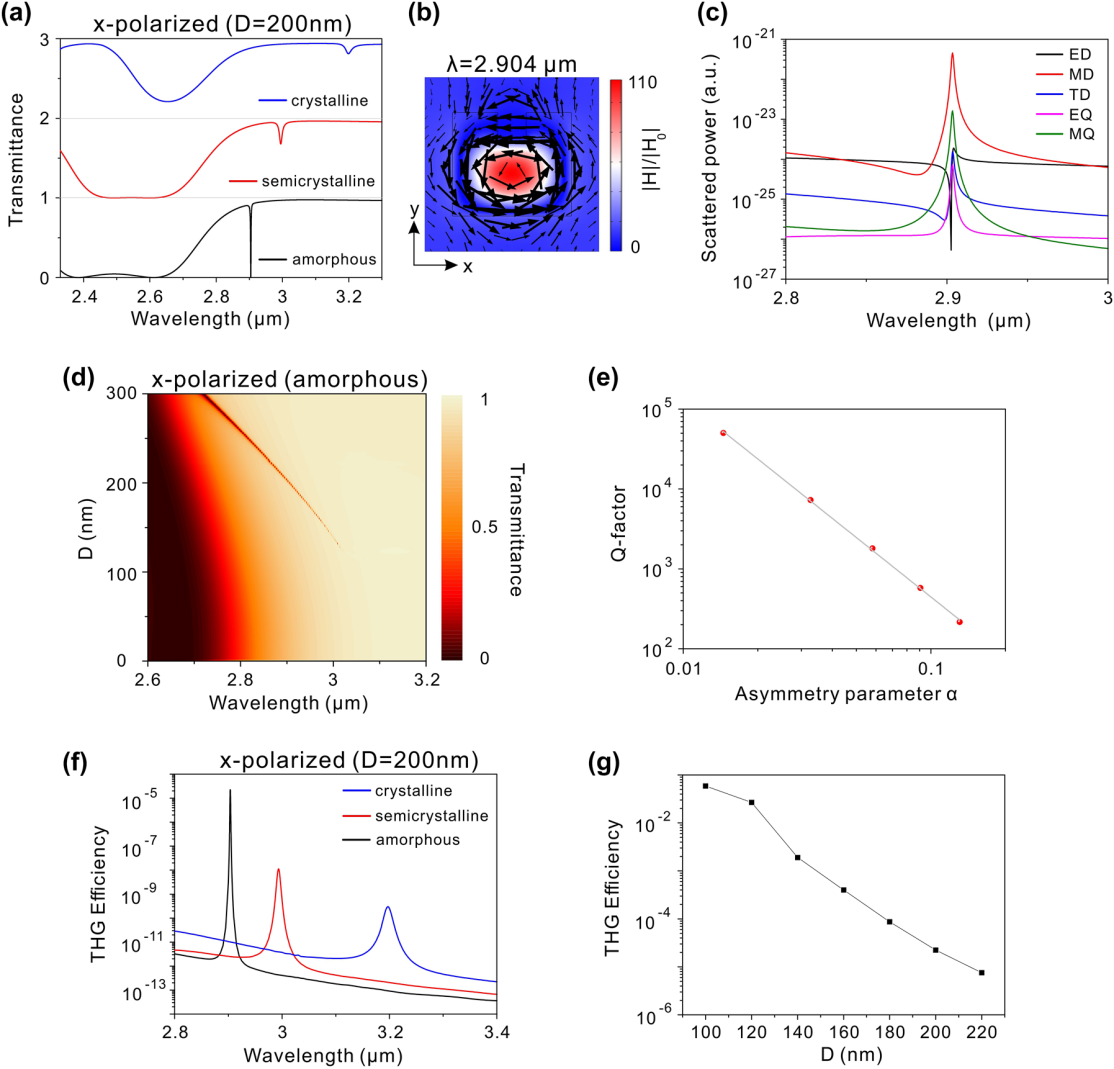
Optical properties of the phase-change metasurface with defect under x-polarized incidence. (a) Transmission spectra of the hybrid phase-change metasurface with *D* = 200 nm at different phases of GST under *x*-polarized incidence. (b) Electromagnetic field distribution at 2.904 μm for GST in the amorphous phase, where the color presents the amplitude of the magnetic field while the arrows indicate the electric field vectors. (c) Cartesian scattered powers of multipole moments for GST in the amorphous phase with *D* = 200 nm. (d) Transmission spectra with different *D* for GST in the amorphous phase under *x*-polarized incidence. (e) Q-factor of the MD quasi-BIC resonance as a function of the asymmetry parameter *α*. (f) THG efficiency as a function of the pump wavelength at different phases of GST with *D* = 200 nm. (g) Dependence of the THG efficiency at MD quasi-BIC wavelength on the parameter *D* for GST in the amorphous phase.

The potential enhancement of THG efficiency through Mie resonances is constrained by the limited electric field enhancement. Recent studies suggest that significant improvements in THG efficiency can be achieved by utilizing high-Q quasi-BICs [13], [14], [15], [16], [17], [18]. As analyzed in Figure 2, the hybrid phase-change metasurface without defect can support two types of BICs. However, these BICs are inaccessible due to the symmetry mismatch. Introducing a defect to break the in-plane inversion symmetry can transform these BICs into quasi-BICs, characterized by high-Q resonances in transmission spectra [19], [20], [21]. Here, a small square defect is incorporated into each Si-GST-Si nanostructure, as depicted in Figure 1(b), with the side length of the square defect denoted as *D* and its height matching the thickness of the Si-GST-Si nanostructure. Initially, we selected *D* as 200 nm and calculated the transmission spectra with various crystallinities of GST under *x*-polarized incidence, as shown in Figure 4(a). In the amorphous phase of GST, a sharp dip emerges at 2.904 μm, exhibiting a Q-factor exceeding 10^3^. As displayed in Figure 4(b), at the wavelength of 2.904 μm, a circular electric field is formed in the *XY* plane and the magnetic field is aligned along the *z*-direction. The electromagnetic field within the nanostructure experiences a significant enhancement compared to the low-Q Mie resonances in Figure 3(b). Additionally, the scattered power of the MD moment predominates at the wavelength of 2.904 μm, as presented in Figure 4(c). Therefore, the sharp dip results from the excitation of *z*-direction MD mode. As GST transitions gradually into the crystalline phase, the *z*-direction MD resonance shifts toward longer wavelengths due to the increased refractive index of GST. However, the linewidth of *z*-direction MD resonance expands during the phase transition, attributed to the increased absorption loss of GST. Figure 4(d) shows the calculated transmission spectra for GST in the amorphous phase with different *D* under *x*-polarized incidence. As *D* decreases gradually, the *z*-direction MD resonance undergoes a redshift. The linewidth of the *z*-direction MD resonance diminishes and vanishes as *D* approaches 0. The retrieved Q-factors of this resonance as a function of the asymmetry parameter are presented in Figure 4(e), where the asymmetry parameter (*α*) is defined as *α* = (*D* × *D*)/(*W* × *W*). It is clear that the Q-factor increases rapidly as *α* decreases and tends toward infinity as *α* approaches 0. Furthermore, the Q-factor exhibits an inverse square relationship with *α*, which is a feature of symmetry-protected BICs [49]. Therefore, the *z*-direction MD resonance is transformed into a BIC mode when *α* is decreased to 0. As discussed in Figure 2, the TE 1 mode in the symmetric metasurface without defect (*α* = 0) is a BIC mode with electromagnetic fields similar to the *z*-direction MD resonance. Hence, the *z*-direction MD resonance is a quasi-BIC mode, originating from the TE 1 mode and induced by the disruption of structural symmetry.

Since a strong electromagnetic field accompanies with the MD quasi-BIC, the THG efficiency can be further enhanced when the fundamental wavelength of pump light is around the wavelength of MD quasi-BIC. Figure 4(f) presents the calculated THG efficiency at different crystallinities of GST, with the pump light polarized along *x*-direction and *D* set to 200 nm. It is observed that in the amorphous phase of GST, the THG efficiency exhibits a sharp peak at 2.904 μm, coinciding with the resonant wavelength of the MD quasi-BIC in the transmission spectrum. The THG efficiency at 2.904 μm is 2.2 × 10^−5^, which is six orders of magnitude higher than that of the ED mode, indicating a significant enhancement in THG efficiency facilitated by the high-Q MD quasi-BIC. After the GST phase transition, the redshift of the MD quasi-BIC leads to a corresponding redshift in the THG peak, as depicted in Figure 4(f). However, as GST gradually transitions into the crystalline phase, the THG peak efficiency diminishes. Since the absorption loss of GST increases during the phase transition, the electric field intensity of the MD quasi-BIC decreases, leading to the reduction in the THG peak efficiency. Consequently, a wavelength-tunable THG enhancement can be achieved through the phase transition of GST. As the Q-factor of MD quasi-BIC can be adjusted by varying the parameter *D*, we also discuss the impact of the parameter *D* on the THG efficiency. Figure 4(g) illustrates the variation of the THG efficiency at the wavelength of MD quasi-BIC with respect to the parameter *D* when GST is in the amorphous phase. Notably, as *D* decreases gradually, the THG efficiency experiences a significant increase, with a THG efficiency of 0.059 reached at *D* = 100 nm. Therefore, maximizing THG efficiency can be achieved by minimizing the size of *D*.

**Figure 5:**
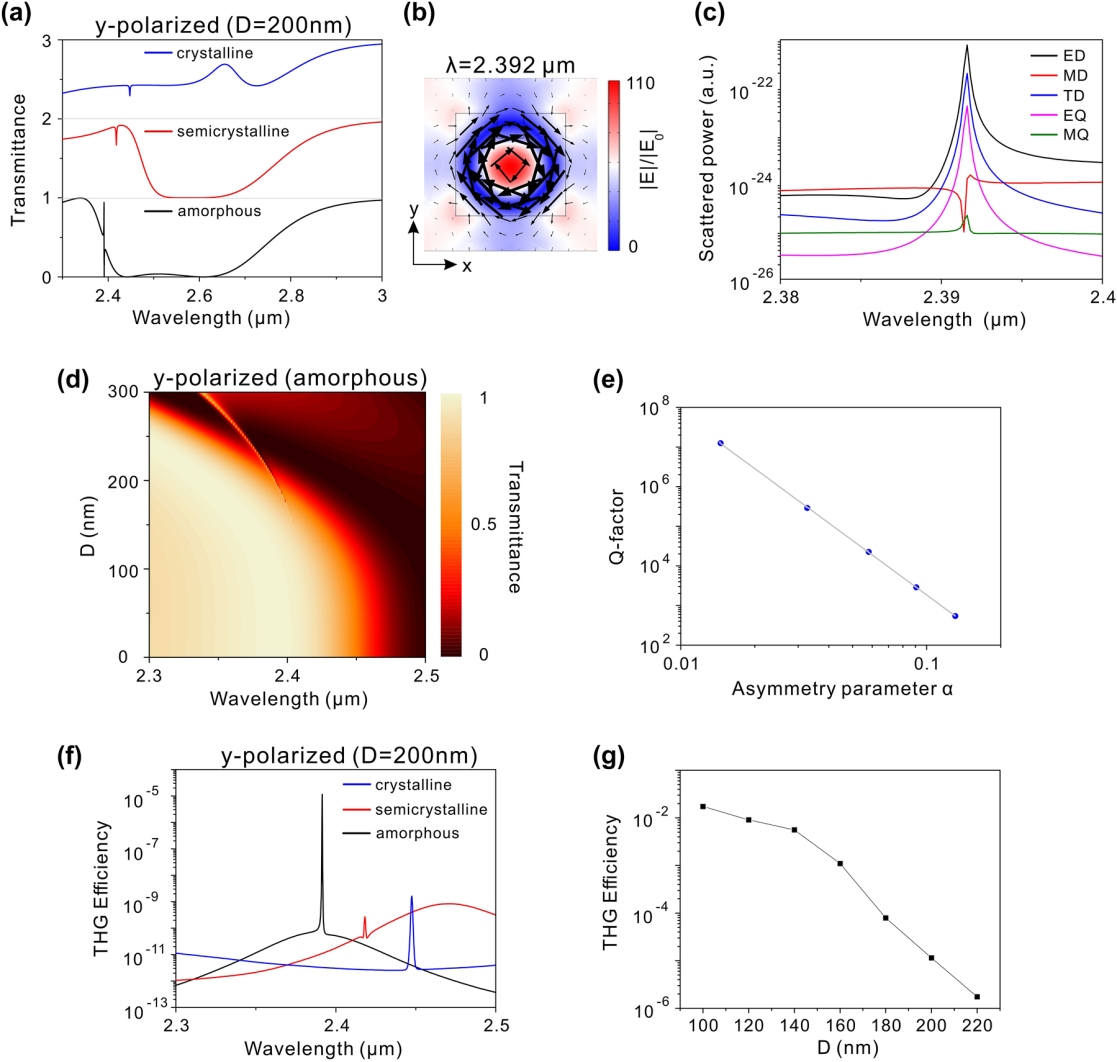
Optical properties of the phase-change metasurface with defect under *y*-polarized incidence. (a) Transmission spectra of the hybrid phase-change metasurface with *D* = 200 nm at different phases of GST under *y*-polarized incidence. (b) Electromagnetic field distribution at 2.392 μm for GST in the amorphous phase, where the color represents the amplitude of the electric field while the arrows indicate the magnetic field vectors. (c) Cartesian scattered powers of multipole moments for GST in the amorphous phase with *D* = 200 nm. (d) Transmission spectra with different *D* for GST in the amorphous phase under *y*-polarized incidence. (e) Q-factor of the ED quasi-BIC resonance as a function of the asymmetry parameter *α*. (f) THG efficiency as a function of the pump wavelength at different phases of GST with *D* = 200 nm. (g) Dependence of the THG efficiency at ED quasi-BIC wavelength on the parameter *D* for GST in the amorphous phase.

In addition to *x*-polarized incidence, we also investigate the optical characteristics of the hybrid phase-change metasurface under *y*-polarized incidence. Figure 5(a) shows the transmission spectra with various crystallinities of GST under *y*-polarized incidence, with a selected value of *D* at 200 nm. In the amorphous phase of GST, the MD quasi-BIC cannot be induced under this polarization; however, a new resonance emerges near 2.392 μm with a Q-factor exceeding 10^4^. At the wavelength of 2.392 μm, the electric field is intensified and along the *z*-direction, accompanying by a circular magnetic field in the *XY* plane, as illustrated in Figure 5(b). Moreover, the scattering power at this wavelength is primarily governed by the ED moment, as shown in Figure 5(c). Consequently, the high-Q resonance originates from the excitation of *z*-direction ED mode. The *z*-direction ED resonance experiences a redshift after the phase transition of GST. Figure 5(d) presents the calculated transmission spectra with different *D* under *y*-polarized incidence for GST in the amorphous phase. As *D* decreases, the *z*-direction ED resonance redshifts while the linewidth reduces and disappears. Additionally, the Q-factor of the *z*-direction ED resonance approaches infinite as *α* is close to 0, and it exhibits an inverse square relationship with the asymmetry parameter, as depicted in Figure 5(e). Consequently, the *z*-direction ED resonance transforms into a BIC mode when the metasurface is symmetric without defect. As analyzed in Figure 2, the TM 1 mode in the symmetric metasurface is a BIC mode with the electromagnetic field similar to the *z*-direction ED resonance. Hence, the *z*-direction ED resonance is a ED quasi-BIC sourced from the TM 1 mode. It should be noted that the MD quasi-BIC is excited in the same structure when the incident light is polarized along the *x*-direction, as discussed in Figure 4. Therefore, the introduction of a defect can lead to the presence of two quasi-BICs with different electromagnetic origins, selectively excited by controlling the polarization of incident light. The calculated THG efficiencies under *y*-polarized incidence are shown in Figure 5(f), with *D* set at 200 nm. In the amorphous phase of GST, a notable THG enhancement is observed at 2.392 μm, consistent with the wavelength of the ED quasi-BIC. Furthermore, the THG efficiency can be further enhanced by reducing the parameter *D*, as illustrated in Figure 5(g). As GST gradually transitions into the crystalline phase, the THG peak shifts to longer wavelength due to the redshift of the ED quasi-BIC. Consequently, wavelength-tunable THG enhancement can be achieved under *y*-polarized incidence. Notably, the MD quasi-BIC can be induced in the same metasurface under *x*-polarized incidence, and THG signal is enhanced at the fundamental wavelength of the MD quasi-BIC, as previously discussed in Figure 4. Given the different wavelengths for the ED and MD quasi-BICs, multiple enhanced THG signals can be obtained in a single metasurface by manipulating the polarization of the pump light and the phase of GST.

**Figure 6:**
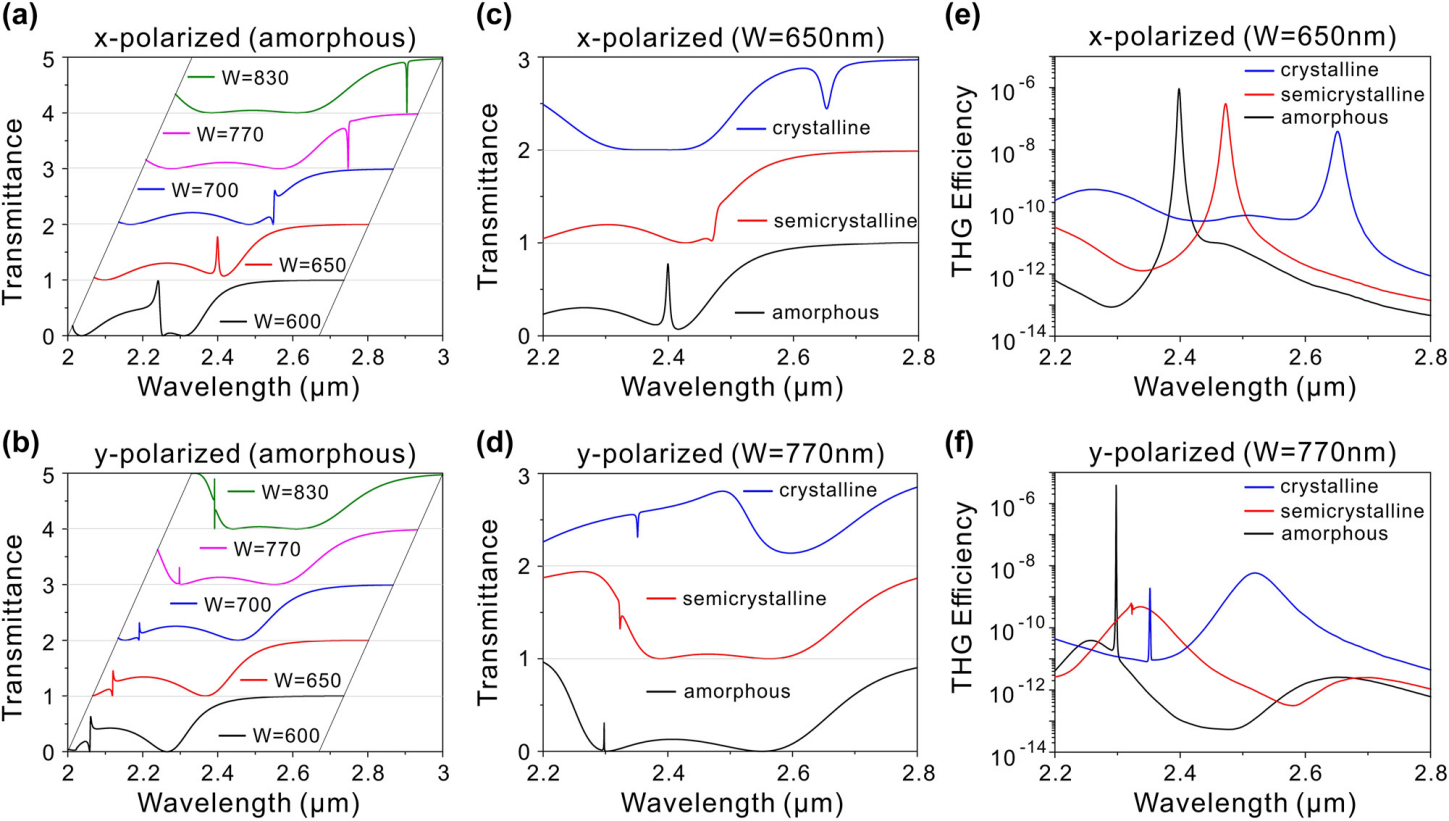
Influence of the side length *W* on the optical properties of the phase-change metasurface. (a) and (b) Transmission spectra of the hybrid phase-change metasurface with different *W* for GST in the amorphous phase under *x*- and *y*-polarized incidence, respectively. (c) Transmission and (e) THG efficiency spectra at different phases of GST with *W* = 650 nm under *x*-polarized incidence. (d) Transmission and (f) THG efficiency spectra at different phases of GST with *W* = 770 nm under *y*-polarized incidence.

Besides the size of defect, we also discuss the effect of the side length *W* on the optical characteristics of the hybrid phase-change metasurface. The transmission spectra under *x*-polarized incidence are depicted in Figure 6(a) for various values of *W*, with GST in the amorphous phase and other geometric parameters the same as those in Figure 4(a). It is observed that all resonances exhibit a blueshift as the *W* decreases, with the MD quasi-BIC displaying a larger wavelength shift compared to the low-Q MD resonance. Notably, when the width is reduced to 650 nm, the MD quasi-BIC couples with the low-Q MD mode, producing an EIT-like phenomenon around 2.4 μm with a Q-factor of 423. However, as GST undergoes a phase transition, the wavelength shift of the MD quasi-BIC surpasses that of the low-Q MD, causing the two resonances to gradually separate, and leading to the disappearance of the EIT phenomenon, as displayed in Figure 6(c). Consequently, a switchable EIT can be realized using the hybrid phase-change metasurface. Interestingly, the EIT phenomenon can also be generated under *y*-polarized incidence by adjusting the parameter *W*, as shown in Figure 6(b). When the width is chosen as 770 nm, an EIT resonance emerges at 2.298 μm due to the coupling between the ED quasi-BIC and low-Q ED mode. Moreover, the EIT resonance can also be switched off by the phase transition of GST, as presented in Figure 6(d). As a result, two types of EIT based on distinct coupling mechanisms can be produced under *x*- and *y*-polarized incidence. Figure 6(e) shows the calculated THG efficiency at different crystallinities of GST, with *W* set at 650 nm and the pump light polarized along the *x*-direction. In the amorphous phase of GST, the THG efficiency reaches a maximum at 2.4 μm, corresponding to the wavelength of EIT resonance. Hence, enhanced THG can be achieved based on the EIT effect. As GST gradually transitions into the crystalline phase, although the EIT vanishes due to the separation between the low-Q MD mode and MD quasi-BIC, enhanced THG also occurs at other wavelengths owing to the presence of MD quasi-BIC. Similarly, enhanced THG can be realized utilizing the EIT induced by *y*-polarized incidence and can be tuned through the GST phase transition, as depicted in Figure 6(f). As a result, active EIT effect can be achieved using the hybrid phase-change metasurface, and wavelength-tunable THG enhancement can be realized by the phase transition of GST.

**Figure 7:**
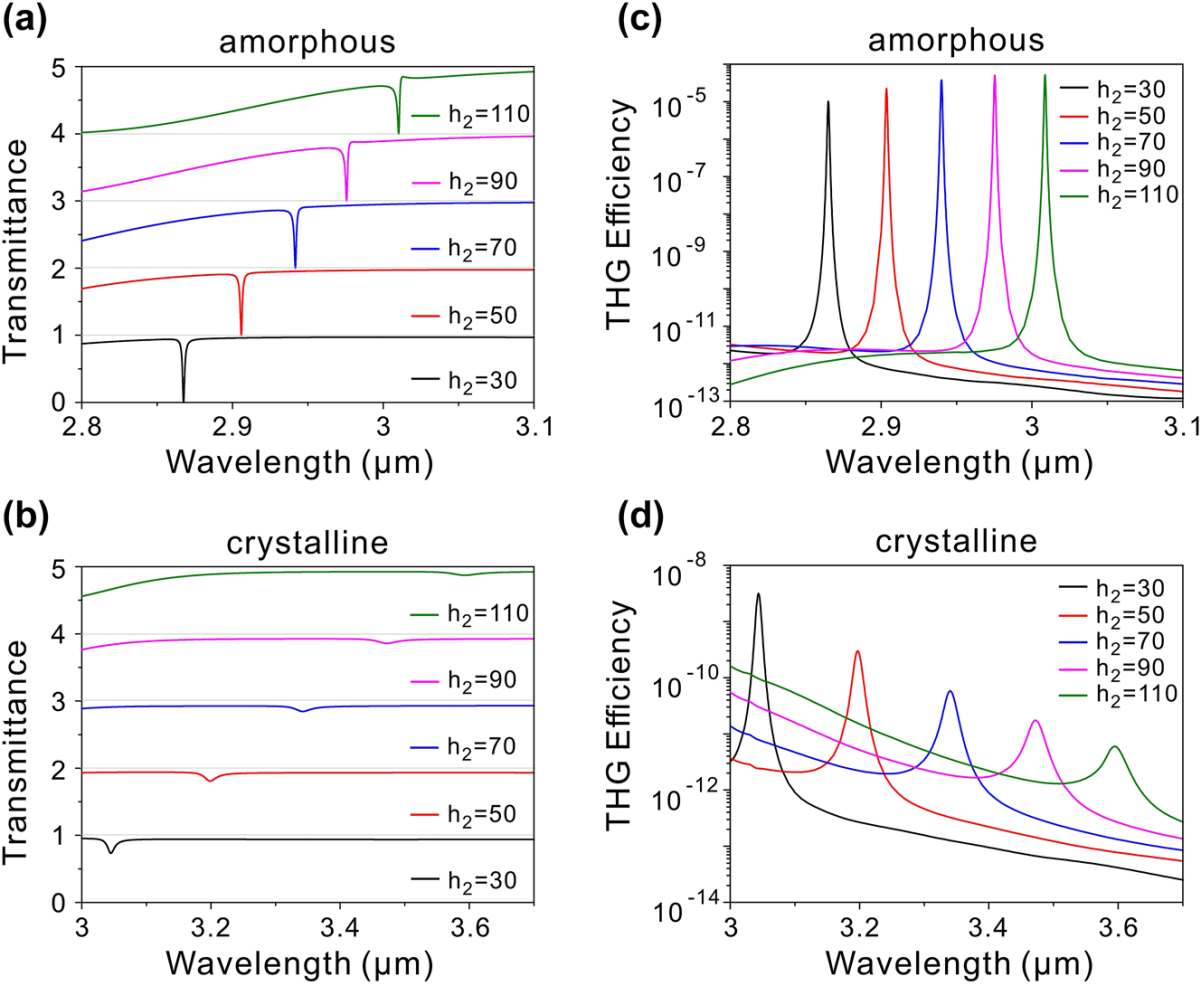
Influence of the GST thickness *h*_2_ on the optical properties of the phase-change metasurface. (a) and (b) Transmission spectra of the hybrid phase-change metasurface with different GST thickness *h*_2_ under *x*-polarized incidence for GST in the amorphous and crystalline phases, respectively. (c) and (d) THG efficiency spectra with different GST thickness *h*_2_ for GST in the amorphous and crystalline phases, respectively, where the pump light is polarized along the *x*-direction.

Finally, we also explore the influence of the GST thickness *h*_2_ on the optical properties of the hybrid phase-change metasurface. Figure 7(a) shows the transmission spectra with varying *h*_2_ under *x*-polarized incidence for GST in the amorphous phase, where the other geometric parameters are the same as those in Figure 4(a). It can be found that the MD quasi-BIC experiences a redshift as *h*_2_ is increased. The redshift in the MD quasi-BIC also leads to a corresponding redshift in the THG peak, as displayed in Figure 7(c). Furthermore, the THG peak efficiency rises with the increase of *h*_2_, attributed to the larger volume of the GST nanostructure. When GST is transmitted into the crystalline phase, the MD quasi-BIC shifts to a longer wavelength due to the increased refractive index of GST, as presented in Figure 7(b). The wavelength shift during the phase transition becomes larger with increasing *h*_2_. In the crystalline phase of GST, the MD quasi-BIC also redshifts with the increase of *h*_2_. However, since the crystalline GST is a lossy material in the considered wavelength range, the linewidth of the MD quasi-BIC expands as the *h*_2_ increases. Figure 7(d) shows the THG efficiency for different *h*_2_ when GST is in the crystalline phase, with the pump light polarized along the *x*-direction. The THG peak undergoes a redshift with increasing *h*_2_, due to the redshift of the MD quasi-BIC. However, the THG peak efficiency reduces as the *h*_2_ increases, owing to the increased absorption loss of GST. Similar variation in the THG efficiency with the GST thickness can also occur when the pump light is polarized along the *y*-direction.

## 4 Conclusions

In summary, we have demonstrated wavelength-tunable THG enhancement using a high-Q polarization-controlled hybrid phase-change metasurface. By introducing a defect to break structural symmetry, a high-Q MD quasi-BIC is excited under *x*-polarized incidence, while a high-Q ED quasi-BIC is induced under *y*-polarized incidence. The THG efficiency is enhanced significantly through the excitation of these quasi-BICs, and the operating wavelength can be tuned by controlling the polarization of pump light. Moreover, the operating wavelength of THG enhancement can be further tuned based on the GST phase transition. Therefore, by manipulating both the polarization of pump light and phase of GST, multiple enhanced THG signals can be generated using a single hybrid phase-change metasurface. Additionally, a high-Q EIT effect can be produced through the coupling between a low-Q Mie resonant mode and a quasi-BIC mode, which also enhances the THG efficiency. The high-Q polarization-controlled hybrid phase-change metasurface sets the stage for the advancement of dynamic nonlinear optical devices.
